# Methylphenidate in Autism Spectrum Disorder: A Long-Term Follow Up Naturalistic Study

**DOI:** 10.3390/jcm9082566

**Published:** 2020-08-07

**Authors:** Patrizia Ventura, Concetta de Giambattista, Laura Spagnoletta, Paolo Trerotoli, Maddalena Cavone, Alessandra Di Gioia, Lucia Margari

**Affiliations:** 1 Child Neuropsychiatric Unit, University of Bari Aldo Moro, Piazza Giulio Cesare, 70124 Bari, Italy; patriziaventuranpi@gmail.com (P.V.); concettadegiambattista@gmail.com (C.d.G.); lauraspagnoletta@hotmail.it (L.S.); magda.cavone@gmail.com (M.C.); aledigioia1993@gmail.com (A.D.G.); 2 Department of Biomedical Sciences and Human Oncology, University of Bari, 70124 Bari, Italy; paolo.trerotoli@uniba.it

**Keywords:** methylphenidate, MPH, autism spectrum disorder, ASD, attention deficit/hyperactivity disorder, ADHD, high-functioning ASD

## Abstract

Autism spectrum disorder (ASD) often co-occurs with attention deficit/hyperactivity disorder (ADHD). Although methylphenidate (MPH) efficacy and safety are well-demonstrated for ADHD, evidences are scant in the context of ASD. This naturalistic study aimed to analyze long-term MPH efficacy and safety in 40 ADHD children and adolescents with comorbid ASD, comparing them with 40 ones affected by ADHD without ASD. Treatment lasted from 6 to 156 months (longer than 24 months in more than three quarters of patients). Efficacy and safety were measured by clinical global impression and children global assessment scales; influence of intellectual functioning was examined. Comparisons between groups were made by Wilcoxon or Friedmann tests; relationships between functioning scores and other characteristics were analyzed by ordinal logistic and linear regression. Results demonstrated that MPH in patients with ASD was associated with significative reduction of illness severity, clinical improvement and amelioration of global functioning, without significant differences with patients having ADHD without ASD. The trend of reduction of illness severity and increase of global functioning were favorably related with intellectual functioning. No serious adverse events were reported. The findings showed that long-term MPH was effective and well-tolerated in ADHD children and adolescents with comorbid high functioning ASD.

## 1. Introduction

### 1.1. ASD and ADHD

Until 2013, autism spectrum disorder (ASD) and attention-deficit/hyperactivity disorder (ADHD) have been both included in the category of neurodevelopmental disorders [1]. ASD is defined by persistent deficits in social interaction and communication as well as restricted, stereotyped and repetitive behaviors and has an overall prevalence between 1 and 2.93% [2,3,4]; whereas ADHD is characterized by inattention and hyperactivity/impulsivity, having a prevalence between 2% and 7% in the pediatric population [5].

Until the fourth revised version of the Diagnostic and statistical manual of mental disorders [6], this one included, diagnosis of ASD in subjects with ADHD has not been allowed, causing clinical and research implications. Instead, in the last version of DSM, ASD and ADHD can be diagnosed as comorbidities, thus they can be studied together. Indeed, also before DSM change, a great amount of studies was performed in order to analyze the potential relation between the two disorders. The co-existence of ASD and ADHD has been reported to be between 25% and 85% [7,8,9,10,11]. This wide range could be justified by both the different nosographic approach used before DSM-5 as well as the clinical difficulties to clearly distinguish the boundaries of each disturbance. Atypical attention patterns, deficit in motor speed, impairment in behavioral inhibition, and social difficulties could be central symptoms of both disorders [12,13,14,15]. Moreover, ASD and ADHD share some common risk factors such as genetics, as found by studies from family, twin and molecular data [16,17,18,19,20]. The shared genetic factors are believed to affect the structure and function of molecular networks in the brain, possibly implicated in the etiology of ASD and ADHD, such as circuits involving executive functions (EF): EF impairment, manifested as deficit in cognitive flexibility, planning, working memory, response inhibition, selective, sustained and shifting attention, has been considered as central etiological deficits in ADHD and recent studies found EF alterations in ASD as well [21,22,23]. Moreover, in the context of co-occurring ASD and ADHD, the functional impairment may be more severe than in the context of ASD alone, since ADHD symptoms may cause obstacles to educational achievement and behavioral management, and since higher risk for psychopathologies such as anxiety and depression may be found [24,25,26,27,28]. As a result, recognizing the comorbidity between ASD and ADHD or the symptoms overlap is crucial for treatment implications.

### 1.2. Psychotropic Drugs in ASD

Epidemiological studies suggested that at least 50% of subjects with ASD receives psychotropic medication during their whole lifespan, and, in pediatric population, the range varies from about 10% to 30% [29,30,31,32].

Indeed, no psychotropic medications have been currently approved by any regulatory agencies for the treatment of ASD core symptom, even if thousands of clinical trials are ongoing [33,34]. For the treatment of associated symptoms, a psychopharmacological approach (antipsychotics, serotonergic agents, stimulants, melatonin) may be beneficial, but data on this topic are not conclusive [10,29,35,36,37,38,39,40,41]. Among second generation antipsychotics (SGA), risperidone and aripiprazole are approved by the Food and Drug Administration for the treatment of irritability associated with ASD, and risperidone has been approved for the same purpose by the European medicines agency. Both have shown modest efficacy for the management of repetitive behaviors, although their use is limited by the risk of adverse effects, especially extrapyramidal and metabolic. Serotonergic agents (SSRIs) are used to treat depression and obsessive-compulsive disorder comorbid with ASD and have shown some benefit for the management of repetitive behaviors and anxiety. Melatonin has demonstrated to be an effective and well-tolerated drug in specifically treating sleeping problems in children with ASD.

Among ADHD targeting drugs in ASD (stimulants, norepinephrine reuptake inhibitors and alpha-agonist agents), the main evidences support the use of MPH; atomoxetine and α2A receptor agonists (clonidine and guanfacine) should be considered as alternatives among those who are not responsive or intolerant to MPH, even if both showed lower efficacy [42,43,44,45].

Despite the common clinical use of psychotropic medications, several reasons underscore the need for caution and additional evidences; in particular, concerning stimulants’ efficacy and safety in ADHD children and adolescents with comorbid ASD, mixed and not conclusive results have been reported from previous studies.

### 1.3. MPH in ADHD with Comorbid ASD

Early studies reported that methylphenidate (MPH) in ASD had modest efficacy, poor tolerability and worsening of behavioral and social problems: Campbell in 1975 noted increased irritability, aggressiveness and stereotypical movements [46]; case reports in 1980s confirmed the worsening of behavioral problems, describing agitation, stereotypes and induction of psychotic symptoms [47,48,49,50]. Following literature, in 1990s and 2000s, has provided additional, but divergent insights both about adverse events and favorable effects, since studies did not find increased irritability nor aggressive behaviors and stereotypes, but rather an improvement in impulsivity and hyperactivity as well as in joint attention, social communication and self-regulation [51,52,53,54,55,56,57,58,59].

These findings were confirmed and enriched by review studies [40,60] comparing MPH-derivatives to placebo in children with ASD. Reichow reviewed four double-blinded, randomized, placebo-controlled trials, involving 94 participants [51,52,56,61]; the Cochrane database included four crossover studies, involving 113 children aged 5 to 13 years [51,52,58,61]. Globally, reviews show that MPH was superior to the placebo for the treatment of hyperactivity and mildly superior for inattention, irritability and stereotypes. There was no evidence that MPH worsened or improved the core symptoms of ASD. In both reviews, the main adverse event reported was decreased appetite. No trials reported serious adverse events, even if authors concluded that the evidence for adverse events had a poor quality, since trials were short and with small sample size.

In 2017, Kim [62] reported significant decrease of irritability and hyperactivity, substantial clinical global improvement, reassuring safety data and low discontinuation rate thanks to extended-release liquid formulation of MPH; nevertheless, the number of subjects was 27 and the treatment duration was 6 weeks.

Considering the common co-occurrence of ASD and ADHD, the current lack of ASD-targeted medications, the misuse of antipsychotics being weighted by adverse events, as well as the well-demonstrated efficacy and safety of MPH (both short and long-term treatment) in ADHD [63,64,65], it could be reasonable to question if a routine use of stimulants for the treatment of ADHD symptoms in the context of ASD is enough supported by evidences.

We aimed to assess, in a naturalistic setting, long-term efficacy and safety of MPH, as monotherapy or associated with other psychotropic drugs, in ADHD children and adolescents with comorbid ASD, comparing them with an ADHD control group, homogeneous for age and intellectual functioning.

## 2. Materials and Methods

### 2.1. Participants and Procedures

This was a naturalistic study based on a clinical database of Caucasian young people, aged between 6 and 21 years, consecutively referred to Child Neuropsychiatric Unit of the University of Bari during a three years period (March 2017–March 2020). The inclusion criteria were ADHD diagnosis according to DSM-5, pharmacological treatment with immediate release MPH (IR-MPH) or extended release MPH (ER-MPH) and therapy lasting at least six months. The exclusion criteria were syndromic autism and age under 6 years. Given the naturalistic design of the study, patients who already took other pharmacological treatments were not excluded. All the subjects were diagnosed according to clinical judgement from the expert team, composed by child and adolescent neuropsychiatrists and psychologists, specialists in neurodevelopmental disorders. Diagnosis was also supported by standardized diagnostic tools, specific for ADHD and ASD: Revised Conners’ Parent Rating Scale (CPRS-R) [66], Autism Diagnostic Observational Schedule, Second Edition (ADOS-2) [67,68], Autism Diagnostic Interview-Revised (ADI-R) [69] and Autism spectrum Diagnostic Interview (ASDI) [70]. Moreover, all the subjects had their intellectual quotient assessed with Wechsler scales: Wechsler Preschool and Primary Scale of Intelligence-Third Edition (WPPSI-III) [71] and Wechsler Intelligence Scales for Children- Fourth Edition (WISC-IV) [72] or Leiter International Performance Scale-Revised (Leiter-R) [73] in patients with communication impairment and were screened for other psychiatric disorders using medical history. Clinical observation and children behavioral checklist (CBCL) [74]. Among 89 patients who started MPH, 9 were excluded because of syndromic autism or being under 6 years of age. The enrolled sample consisted of 80 patients of which 40 had a diagnosis of ADHD comorbid with ASD (henceforth named ASD + ADHD group) and 40 had a diagnosis of ADHD without comorbidity with ASD (henceforth named ADHD group).

Demographic and clinical characteristic of the two groups, comorbidities and pharmacological treatments beside MPH are described in Table 1.

All the diagnostic and therapeutic procedures, as well as the follow-up and data collection, were part of our standard routine. All subjects and parents received detailed information on the assessment measures and different treatment options and gave their written informed consent to the treatment with MPH. Medication was prescribed according to clinical indications, along with routine community care (including psychoeducational interventions), as part of a management protocol.

The Ethic Committee of the Azienda Ospedaliera-Universitaria Consorziale Policlinico di Bari approved the study (5009/29-07-2016).

The research team reviewed medical records of all the patients for the purpose of the study; in particular, the team extrapolated data concerning efficacy and safety measures, such as scores at clinical global impression scale (CGI) [75] and children’s global assessment scale (C-GAS) [76], as well as anthropometric measures (height and weight), vital parameters (blood pressure, heart rate), blood test reports (blood cell count, blood chemistry, electrolytes, thyroid function) and cardiac assessment (ECG including QTc interval).

CGI is a brief clinician-rated instrument that consists of three different global measures: severity of illness (CGI-S), global improvement (CGI-I) and efficacy index (CGI-E). CGI-S is rated from 1 (normal, not at all ill) to 7 (among the most extremely ill patients). CGI-I is a 7 points scale that requires the clinician to assess how much the patient’s illness has improved or worsened with respect to a baseline state at the beginning of the intervention and is rated from 1 (really improved) to 7 (really worsened). CGI-E is a 4×4 rating scale assessing the therapeutic effect of treatment with psychiatric medication and associated side effects. The patient’s response to treatment is combined with any salient side effect on a grid to give an CGI-E. A child with a good response to treatment and no reported side effects would have an CGI-E of 4.00. A patient with no response to treatment or who got worse during treatment, having significant side effects thus requiring a change in treatment, would have an CGI-E of 0.25.

C-GAS is a numeric scale used by mental health clinicians to rate the general functioning of child and youths under the age of 18. Scores range from 1 to 100, with higher scores indicating better functioning. Patients with a C-GAS ranging from 70 to 61 struggle in a single area, albeit functioning generally well; patients with a C-GAS ranging from 60 to 51 have variable functioning with sporadic difficulties or symptoms in several, but not all social areas; patients with a C-GAS ranging from 50 to 41 have moderate degree of interference in functioning in most social areas or severe impairment of functioning in one area; patients with a C-GAS ranging from 40 to 31 have major impairment of functioning in several areas; patients with a C-GAS ranging from 30 to 21 are unable to function in almost all areas.

Primary outcomes were efficacy and safety data, measured by CGI-S, assessed at the baseline (T0), after 1 month (T1), after 6 months (T6), after 24 months (T24); CGI-I, assessed after 1 month (T1), after 6 months (T6), after 24 months (T24); CGI-E, assessed at the last follow-up and C-GAS—assessed at the baseline (T0) and at the last follow-up. Safety data were analyzed at baseline and at each follow-up. Discontinuation rate was recorded and described.

Secondary outcome was the influence of different variables (intellectual functioning, age of therapy onset and length of therapy, dose of drugs, sex and age) on efficacy (measured by CGI-S and CGI-I) and global functioning (measured by C-GAS).

All data obtained from ASD + ADHD group and ADHD group were compared to each other, henceforth analyzed.

### 2.2. Statistical Analysis

Quantitative data, as CGI-S, CGI-I, CGI-E, C-GAS, were not normally distributed, therefore the comparison between disease group was performed with Wilcoxon for independent groups. A test for repeated measures, i.e., Friedman test, was performed separately in each disease group, in order to compare results among follow-ups. Given the multiple test approach of the analysis, *p*-values were adjusted according to Bonferroni. Quantitative variables were summarized as median, range and interquartile range or 95% confidence interval for the median.

Qualitative variables are summarized as count and percentage, comparisons between independent groups were performed by χ^2^ test or Fisher’s exact test when appropriate. In order to compare the percentages of side effects, a multiple comparison strategy was applied: the statistical Fisher’s exact test was used, and *p*-values were adjusted according to a permutation method for the purpose of controlling family wise error rate.

An ordinal logistic model was performed to evaluate predictors of changes in CGI-I and CGI-S.

The model for CGI-S had ΔCGI-S as dependent variable, defined as the difference obtained subtracting the CGI-S score at T24 from the CGI-S score at T0, classified as neutral (0) and classes of increasing improvement (from −1 to −3).

The model for CGI-I had a dependent variable classified as improved if the score decreased, neutral if there was no difference between CGI-I score at T1 and CGI-I score at T24, and not improved if the score increased.

The predictors in both models were: age, sex (as dummy: M = 1, F = 0), length of therapy (in months), age of therapy onset, dose of drug, disease group (as dummy: ADHD = 1, ASD + ADHD = 0). A backward selection procedure was used for the multivariable model, but, in order for variables to have an effect independently from the disease group, a new model was fitted including this variable; R-square was used as measure of fitting.

A *p*-value < 0.05 was used to assess statistical significance.

A multiple linear regression was used to evaluate if changes in C-GAS depend on IQ and other possible predictors (disease group, age, sex, age of therapy onset, dose of drugs, length of therapy). A backward selection method was applied with a *p*-value < 0.1 as threshold to enter the model, in order to choose best predictors.

The software used for the analysis was SAS 9.4 (SAS Institute, Inc., Cary, NC, USA).

## 3. Results

### 3.1. MPH Treatment

The starting dose of MPH was 0.3–0.5 mg/kg/day. The dosage could be increased up to 1 mg/kg/day depending upon the subject’s clinical response and tolerability, up to maximum of 60 mg/day. The total dose could be administered in two or three doses/day. After one month of titration, the IR-MPH was generally replaced with ER-MPH. The range of therapy duration was from 6 to 156 months. A total of 18/80 participants (22.5%) of the total sample had a follow-up treatment lower than 24 months, 22/80 participants (27.5%) had a follow-up treatment ranging from 24 months and 36 months, 40/80 participants (50%) had a follow-up treatment higher than 36 months. At the last follow-up, 73/80 participants (91.25%) were treated with ER-MPH (37/40 ASD + ADHD subjects and 36/40 ADHD subjects); 7/80 participants (8.75%) were treated with IR-MPH (3/40 ASD + ADHD subjects and 4/40 ADHD subjects). Characteristics of MPH treatment were described in Table 2.

### 3.2. Primary Outcomes

#### 3.2.1. CGI Measures 

In ASD + ADHD group the difference in CGI-S from T0 to T24 was statistically different (*p* < 0.0001) Comparing the ASD + ADHD group with the ADHD group, the difference in CGI-S improvement was not statistically significant in none of the follow-up points. Moreover, 62,2% (23/36) in ASD + ADHD group and 79.5% (31/39) in ADHD group has shown a ΔCGI-S (from T0 to T24) lower or equal to −2, which is a fairly good amelioration, in both groups, without statistically significant difference (*p* = 0.3255).

In ASD + ADHD group the difference in CGI-I from T0 to T24 was statistically different (*p* = 0.0001). Comparing the ASD + ADHD group with the ADHD group, the difference in CGI-I was not statistically significant in none of the follow-up points. The percentage of responders (patients with a CGI-I T24 score 1, “very much improved” and score 2, “much improved”) was 70.3% (26/37) in ASD + ADHD and 64.1% (25/39) in ADHD patients, but the difference was not statistically significant (*p* = 0.5699).

In ASD + ADHD group the median (95% CI) CGI-E was 2 (1.5–2). Comparing the ASD + ADHD group with the ADHD group, the difference in CGI-E was not statistically significant. CGI measures are described in Table 3.

#### 3.2.2. C-GAS Measures 

In ASD + ADHD group the difference in C-GAS from T0 to the last follow-up was statistically different (*p* < 0.0001) Comparing the ASD + ADHD group with the ADHD group, the difference in C-GAS was not statistically significant at either T0 or last follow-up. C-GAS measures are described in Table 4.

#### 3.2.3. Other Psychotropic Medications at the Last Follow-Up

At the last follow-up, in ASD + ADHD group, 7.5% of patients assumed antipsychotics (second generation; 27.5% at baseline), 7.5% mood stabilizers (10% at baseline), 2.5% antidepressants (5% at baseline), 2.5% anxiolytics and 20% melatonin (both equal to baseline); in ADHD group, 12.5% of patients assumed antipsychotics (second generation; 17.5% at baseline), 10% mood stabilizers (12.5% at baseline), 2.5% antidepressants and 22.5% melatonin (both equal to baseline).

#### 3.2.4. Safety 

No severe adverse events were reported; indeed, among these, no cardiovascular events neither suicidal ideations nor behaviors were seen.

Comparing ASD + ADHD group with ADHD group, the prevalence of the lack of side effects was not statistically significant (respectively 29/40, 72.5%; 27/40, 67.5%; *p* = 0.6256) 

The most frequent side effects in ASD + ADHD group were loss of appetite, abdominal discomfort and headache (respectively 47.5%, 45% and 25%), each temporary, in the first days or weeks of treatment; the same side effects were seen in ADHD group (respectively in 57.7%, 35% and 15%).

Two ADHD patients with comorbid ASD (5%), both affected by level 3 ASD associated with intellectual disability, after partially responding to the treatment for few weeks, presented worsening of behavior with restlessness and increased stereotypes, that completely resolved after treatment discontinuation. In the ADHD group, one patient (2.5%), affected by anxiety disorder, had to stop the drug, despite it worked on attention, due to the onset of restlessness and a state of “inner tension”, that resolved once stopping the medication. Side effects are described in Table 5.

#### 3.2.5. Discontinuation Rate

In ASD + ADHD group, eight patients interrupted MPH treatment (20%) for the following reasons: four patients because of clinical improvement (10%); two patients (both of level 3 of severity and intellectual disability) because of clinical worsening (5%); one patient because of low compliance to the treatment (2.5%); one patient was lost at follow-up (2.5%).

In ADHD group, nine patients interrupted MPH treatment (22.5%) for the following reasons: clinical improvement for six patients (15%); clinical worsening for one patient (2.5%); low compliance to the treatment for one patient (2.5%); one patient was lost at follow-up (2.5%).

The discontinuation rate did not result significantly different (χ^2^ = 0.075, *p* = 0.7846).

### 3.3. Secondary Outcomes

#### 3.3.1. Evaluation of Factors to Predict Amelioration in Severity of the Illness

**Figure 1:**
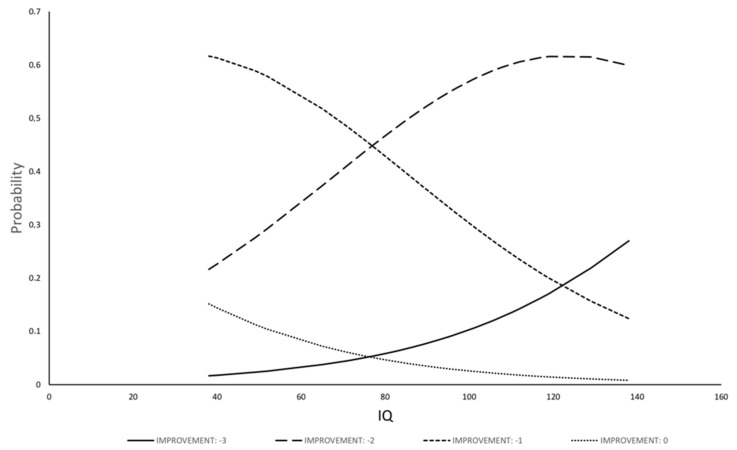
Relationship between probability to have a certain class of ΔCGI-S amelioration and IQ in ASD + ADHD group.

**Figure 2:**
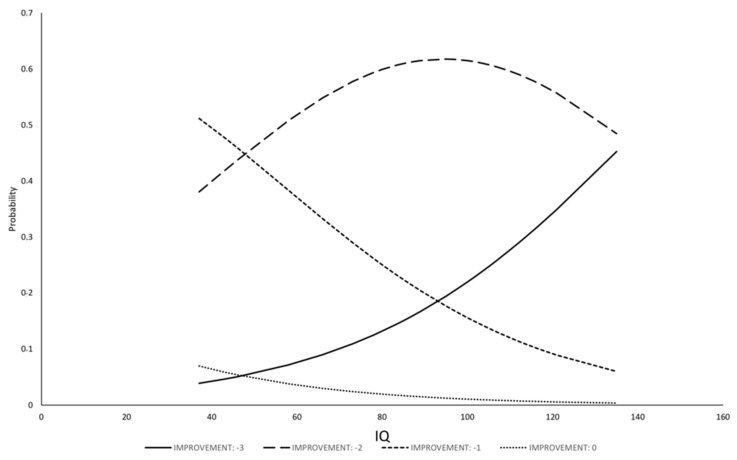
Relationship between probability to have a certain class of ΔCGI-S amelioration and IQ in ADHD group.

The model for ΔCGI-S has shown that IQ was the only statistically significant variable related to changes in severity of illness; other variables (sex and age; disease groups; age of therapy onset; length of therapy; dose of drug) were removed from the model by the selection procedure. The regression for ΔCGI-S was fitted with IQ (b = 0.031, Se(b) = 0.01, *p* = 0.0094), the model resulted statistically significant (likelihood 10.74, *p* = 0.0046) and the R-square of the model was 13.2%. The model allows to estimate the probability of each class of improvement as function of IQ (Figure 1 and Figure 2): the probability of no improvement or of low-class improvement decreases with the increase of IQ; the probability of higher class of improvement, on the contrary, increases with IQ.

#### 3.3.2. Evaluation of Factors to Predict Clinical Improvement

The model for CGI-I has shown that statistically significant variables related to changes in clinical improvement were age (b = −0.23, Se(b) = 0.09, *p* = 0.0094) and age of therapy onset (b = 0.21, Se(b) = 0.09, *p* = 0.021). Coefficients suggest that the elder the patient the lower the probability of the improvement and the earlier starting treatment the higher the probability of improvement. The disease group was not statistically significant (b = −0.05, Se(b) = 0.23, *p* = 0.8356) and also the other variables were removed from the model because not being statistically significant and having a false increase in R-square measure, which was 9.6% in the final model.

#### 3.3.3. Evaluation of Factors to Predict Amelioration in Global Functioning

**Figure 3:**
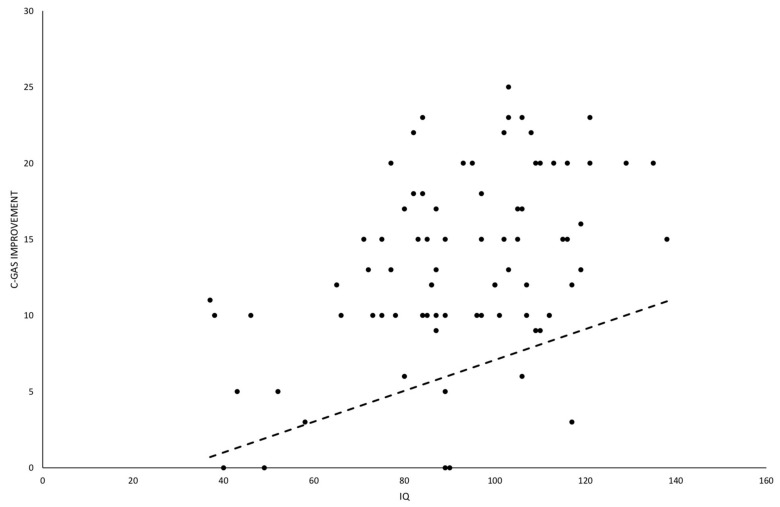
Scatter plot to evaluate relationship between C-GAS improvement and IQ. This factor resulted statistically significant in the multivariable model. The line is drawn with the slope (b = 0.1, *p* = 0.0003) estimated in the multivariable model and other variables set at their mean level.

A statistically significant effect on amelioration of C-GAS by IQ (b = 0.1, *p* = 0.0003), dose of drug (b = 6.3, *p* = 0.0065), sex (dummy 1 = male, 0 = female; b = −2.8, *p* = 0.0446) and age (b = 0.38, *p* = 0.0231) was found. The results of the regression, having an R-square = 32%, suggested that C-GAS increased in higher IQ and for higher dose, but it decreased in male subjects. The disease group was not statistically significant (b = −0.29, *p* = 0.8030) and also the other variables (length of therapy, age of starting treatment) did not result statistically significant. The effect of IQ could be described by a line with the slope obtained by the model that shows how improvement of C-GAS increases when IQ is higher (Figure 3). It should be noticed that IQ gives a partial prediction, because the predicted value depends upon all factors entered in the model.

## 4. Discussion

To date, no medications have been approved for the clinical use specifically for ASD, but, in clinical settings, psychotropic drugs were largely prescribed for comorbid symptoms. Stimulants were among the most prescribed medications: a large study of 2800 children recruited from the Autism Treatment network in North America displayed that stimulants were often prescribed in ASD (13%), followed by SSRIs (8%) and SGA (8%) [30]; data from a study in the United Kingdom, using a representative primary care database, revealed that psychotropic drugs are prescribed to about a third of children and adolescents with ASD and found sleep medications (9.7%), stimulants (7.9%) and antipsychotics (7.3%) to be the most commonly prescribed categories of drugs for ASD [32]; a recent study, based on the Danish national database registry, described psychotropic drugs prescription trends from 2010 to 2017 in 23,935 ASD children and adolescents, born between 1992 and 2011, confirming that 30% of the sample used psychotropics, most commonly ADHD medications (17%) and melatonin (13%), followed by antipsychotics (5%) and antidepressants (5%) [29].

As a matter of fact, the history concerning the co-presence of ASD and ADHD, as well as the consequent application of stimulants also for autistic subjects, has been marked by limits and ambivalences that delayed diagnostic and therapeutic progress. Nowadays, in scientific community, the comorbidity between ASD and ADHD is judged as a common feature and stimulants application emerges as a prolific research field. Considering the clinical traits and neurobiological deficits shared by ASD and ADHD, it has been possible to speculate that subjects with ASD may benefit from the same evidence-based pharmacological treatment, successfully used in subjects with ADHD without ASD.

The first findings have shown that methylphenidate (MPH) was less efficacious and tolerated in people with ASD, compared to ADHD alone [46,47,48,49,50]. Following research found different results, better for efficacy and safety than previous ones [51,52,53,54,55,56,57,58,59]. Nevertheless, both case report and following reviews [40,60] were not conclusive and poorly significant, due to the small number of studies which all had small sample size, poor clinical characterization and short-term follow-up.

The present study analyzed long-term MPH efficacy and safety in a naturalistic setting of 40 ADHD patients with comorbid ASD, compared to 40 patients with ADHD. All the participants had a follow-up lasting from 6 to 156 months (median of therapy duration: 36 months in ASD and ADHD; 48 months in ADHD) of treatment, strictly clinically characterized and monitored.

Results suggested that MPH in ADHD children and adolescents with comorbid ASD was associated with significative reduction of illness severity, clinical improvement in the first two years of treatment and amelioration of global functioning until the last follow-up, without significant differences with children and adolescents with ADHD without ASD at one, six and twenty-four months follow-up.

The present study also found a higher probability of clinical improvement in subjects from both groups who start an earlier MPH treatment. Furthermore, by analyzing the influence of intellectual functioning, results demonstrated a favorable trend of reduction of severity of illness and amelioration of global functioning associated with higher IQ. These findings are in accordance with previous studies [58], in which high functioning ASD were more likely to have a favorable response to MPH treatment than low functioning ones. As for MPH efficacy in ADHD without ASD, considering the robust literature, the influence of intellectual functioning seems controversial. The study from Grizenko et al. (2012) [77], about 502 children with ADHD treated with MPH, showed that there was no statistically significant difference in the response of MPH for children in the border-line, average and superior IQ levels. Tarrant et al. (2018) [78] reviewed studies including 315 participants in order to compare the effectiveness of MPH in typically growing children and adolescents with an intellectual disability (ID). This review showed that the ES of MPH in ID children was lower than that in the non-ID children (0.5 vs 0.8–1.3); type and rate of adverse effects among the two groups seem similar [78].

As for safety data, treatment with MPH in ASD patients was well-tolerated. There were no serious adverse events. The main adverse events were temporary loss of appetite, abdominal discomfort and headache, without significant differences with ADHD group. Only two ADHD patients with comorbid ASD (5%) presented major adverse events consisting in worsening of behavior with restlessness and increased stereotypes that caused the interruption of treatment and both resolved completely after treatment discontinuation; interestingly, both patients were affected by level 3 ASD associated with intellectual disability.

The mean of efficacy index, measured by dividing therapeutic effect score by side effect score, was indicative of a marked therapeutic effect with no significant interference of side effects, or, alternatively, of a minimal therapeutic effect without side effects and there were not significant differences among the two groups. Concerning treatment persistence, previous studies revealed that MPH in ASD patients, compared to other classes of psychotropic drugs, showed a lower rate of treatment discontinuation, with a higher adherence rate in children than in adolescents, that may be explained by children’s greater parent involvement in controlling treatment [29,79]. In the present study, the adherence rate was preserved both in children and adolescents by a strictly monitored follow-up, with informative and motivational support to parents and adolescents. Discontinuation rate did not result significantly different in the two groups and it was prevalently motivated by clinical improvement.

Although not being a focus of this study, our results revealed that co-therapy (e.g., stimulants and risperidone or mood stabilizers or antidepressants or melatonin) did not interfere with response rate to MPH or increased side effects, according with other reports [58,62], and, in addition, a decreased trend in the assumption of other psychotropic medications was found. The intake of melatonin did not change in both groups from the baseline to the last follow-up, proving that MPH treatment did not significantly interfere with sleep/wake rhythm.

These reassuring findings about MPH efficacy, safety and treatment persistence were in accordance with literature from 2000s [52,55,58,62,80] and in discord with early studies that found more MPH adverse events in ASD patients than in ADHD patients [46,47,48,49,50]. We hypothesize that these discordant data could be caused by clinical heterogeneity of the recruited samples or by the assumption of other psychotropic drugs. Both Birmaher (1988) [80] and Quintana (1995) [51] have suggested that the increase in stereotypies seen in children reported by Campbell and colleagues may be more related with withdrawal dyskinesia associated with antipsychotic discontinuation rather than with stimulant induced stereotypies. We assume that low functioning ASD could have an increased risk of worsening behaviors and stereotypes, since in our experience we found worsening of these adverse events only in two patients with ASD associated with intellectual disability.

The present study had different strengths: first of all, the sample was phenotypically characterized, throughout intellectual profile, severity specifiers, diagnostic subtypes and comorbidities; patients were strictly monitored and followed at least every six months, the follow-up period being longer than 2 years in over three-quarters, higher than most the other studies; it described both effectiveness of Immediate Release and Extended Release formulation of MPH. Finally, being it a naturalistic study, clinicians could feel confident in titrating medication and adjusting doses as needed, therefore collected data are closer to real-life setting.

The limitations concerned the utilization of global efficacy measures, rather than specific ASD and ADHD measures for core symptoms. These aspects, not deeply investigated in this study, could represent a future direction of research about MPH effect in ASD + ADHD patients.

Compared with previous studies, the present one showed higher response rate and tolerability of MPH treatment in ASD patients. We hypothesized that this improved stimulants’ profile in ASD may be explained, besides the above-mentioned features associated with naturalistic setting, by the high percentage of subjects with average intellectual functioning in the sample.

In conclusion, our study shows that long-term MPH treatment, lasting 24 months on average, is effective and well-tolerated in ADHD children and adolescents with comorbid high-functioning ASD, even in association with other psychotropic drugs. Although long term studies are necessary to confirm these results, we recommend a routine use of MPH in ADHD with comorbid high-functioning ASD.

## Figures and Tables

**Table 1:** Demographic and clinical characteristics of the sample, comorbidities and other psychotropic medications beside methylphenidate (MPH).

	ASD + ADHD Group (*n* = 40)		ADHD Group (*n* = 40)		*p*-Value
	***n***	**%**	***n***	**%**	
**Sex**					
M	32	80	28	70	
F	8	20	12	30	0.3047
	**Median**	**95% CI**	**Median**	**95% CI**	
** Age (years)**	15.5	14–17	15	14–17	0.7502
	***n***	**%**	***n***	**%**	
**ASD level**					
1	34	85			
2	4	10			
3	2	5			
**ADHD severity level**					
Severe	12	30	14	35	0.6352
Moderate	28	70	26	65	
**ADHD specifiers**					
Inattentive	8	20	6	15	0.5587
Combined	32	80	34	85	
	**Median**	**95% CI**	**Median**	**95% CI**	
** Intelligence Quotient (IQ)**	89.5	84.33–101.66	98.5	86.33–104.32	0.4558
	***n***	**%**	***n***	**%**	
**Comorbidities**					
Learning disorder	31	77.5	32	80	
Motor coordination disorder	11	27.5	2	5	
Sleep–wake disorder	10	25	11	27.5	
Anxiety disorder	9	22.5	6	15	
Oppositional defiant disorder	6	15	32	80	
Disruptive mood dysregulation disorder	5	12.5	8	20	
**Other psychotropic medications**					
Antipsychotics (FGA and SGA)	11	27.5	7	17.5	
Mood stabilizer	4	10	5	12.5	
Antidepressants	2	5	1	2.5	
Anxiolytics	1	2.5	0	0	
Melatonin	8	20	9	22.5	

ASD—autism spectrum disorder; ADHD—attention deficit hyperactivity disorder; CI—confidence interval; FGA—first generation antipsychotics; SGA—second generation antipsychotics.

**Table 2:** MPH treatment.

	ADHD + ASD		ADHD		*p*-Value
**Age of therapy onset (Years)**	**Median (IQR)**	**95% CI**	**Median (IQR)**	**95% CI**	0.2309
	9 (7–13)	7–10	10 (8.5–13)	9–11.6	
**MPH dose (mg/kg/day)**	0.6 (0.46–0.7)	0.5–0.7	0.6 (0.46–0.825)	0.52–0.7	0.6026
**Therapy duration (months)**	36 (24–78)	24–78	48 (36–57.97)	24–72	0.5679

ASD—autism spectrum disorder; ADHD—attention deficit hyperactivity disorder; MPH—methylphenidate; IQR—Inter-quantile Range; CI—confidence interval.

**Table 3:** Clinical global impression scale (CGI) measures.

CGI-S	ASD + ADHD Group	ADHD Group	*p*-Value
**T0**	*n* = 40	*n* = 40	
Median Range (95% CI)	5 4–7 (5–6)	5 4–7 (5–6)	0.5609
**T1**	*n* = 40	*n* = 40	
Median Range (95% CI)	5 3–7 (4.5–5)	5 3–6 (4–5)	0.1138
**T6**	*n* = 39	*n* = 40	
Median Range (95% CI)	4 2–7 (4–5)	4 2–6 (3–4)	0.0501
**T24**	*n* = 37	*n* = 39	
Median Range (95% CI)	4 2–5 (3.4–4)	3 2–6 (3–4)	0.1104
***p*-value** (T0–T24)	<0.0001	<0.0001	
**CGI-I**	**ASD + ADHD group**	**ADHD group**	***p*-value**
**T1**	*n* = 40	*n* = 40	
Median Range (95% CI)	3 2–5 (3–3)	3 2–4 (3–3)	0.9715
**T6**	*n* = 39	*n* = 40	
Median Range (95% CI)	3 2–6 (2–3)	2 2–4 (2–3)	0.594
**T24**	*n* = 37	*n* = 39	
Median Range (95% CI)	2 1–4 (2–3)	2 1–4 (2–3)	0.8116
***p*-value** (T1–T24)	0.00001	<0.00001	
**CGI-E**	**ASD + ADHD group**	A**DHD group**	***p*-value**
Median Range (95% CI)	2 0.25–3 (1.5–2)	2 0.5–4 (1.5–2)	0.7986

ADHD—attention deficit hyperactivity disorder; ASD—autism spectrum disorder; CGI-S—clinical global impression-severity scale; CGI—clinical global impression-improvement scale; CGI-E—Efficacy Index; CI—confidence interval; T0—baseline; T1—after 1 month; T6—after 6 months; T24—after 24 months.

**Table 4:** C–GAS measures.

	ASD + ADHD Group	ADHD Group	*p*-Value
**T0** Median Range (95% CI)	43 25–50 (40–45)	45 30–55 (45–47.66)	0.0632
**Last follow-up** Median Range (95% CI)	55 25–70 (50–58.32)	60 40–70 (55–64.32)	0.0907
***p*-value** (T0–T24)	<0.0001	<0.0001	

ADHD—attention deficit hyperactivity disorder; ASD—autism spectrum disorder; C-GAS—children’s global assessment scale; CI—confidence interval; T0—baseline.

**Table 5:** Side effects.

	ASD + ADHD Group *n* (%)	ADHD Group *n* (%)	*p*-Value
Loss of appetite	19 (47.5)	23 (57.5)	0.9865
Abdominal discomfort	18 (45)	14 (35)	0.9792
Headache	10 (25)	6 (15)	0.8147
Irritability	4 (10)	3 (7.5)	1
Palpitation	3 (7.5)	1 (2.5)	0.9974
Restlessness	3 (7.5)	1 (2.5)	0.9974
Anxiety	2 (5)	0	0.97
Insomnia	1 (2.5)	2 (5)	1
Dizziness	1 (2.5)	1 (2.5)	1
Drowsiness	0	0	1
Tic disorder	0	0	1
Hallucinations	0	0	1

ASD—autism spectrum disorder; ADHD—attention deficit hyperactivity disorder.
